# Vitamin D and Swimming Exercise Prevent Obesity in Rats under a High-Fat Diet via Targeting FATP4 and TLR4 in the Liver and Adipose Tissue

**DOI:** 10.3390/ijerph192113740

**Published:** 2022-10-22

**Authors:** Eman Kolieb, Shymaa Ahmed Maher, Mohammed Nader Shalaby, Amnah Mohammed Alsuhaibani, Afaf Alharthi, Wael A. Hassan, Karima El-Sayed

**Affiliations:** 1 Medical Physiology Department, Faculty of Medicine, Suez Canal University, Ismailia 41522, Egypt; 2 Medical Biochemistry and Molecular Biology Department, Faculty of Medicine, Suez Canal University, Ismailia 41522, Egypt; 3 Center of Excellence in Molecular and Cellular Medicine (CEMCM), Faculty of Medicine, Suez Canal University, Ismailia 41522, Egypt; 4 Biological Sciences and Sports Health Department, Faculty of Physical Education, Suez Canal University, Ismailia 41522, Egypt; 5 Department of Physical Sport Science, College of Education, Princess Nourah bint Abdulrahman University, P.O. Box 84428, Riyadh 11671, Saudi Arabia; 6 Department of Clinical Laboratory Sciences, College of Applied Medical Sciences, Taif University, P.O. Box 11099, Taif 21944, Saudi Arabia; 7 Department of Pathology, Faculty of Medicine, Suez Canal University, Ismailia 41522, Egypt; 8 Department of Basic Sciences, College of Medicine, Sulaiman Alrajhi University, Al Bukayriyah 52726, Saudi Arabia

**Keywords:** HFD: (high-fat diet), obesity, Exercise Physiology, Sports Exercise, Swimming Exercise Vit D: (vitamin D), FATP4: (fatty acid transport protein 4), TLR4: (Toll-like receptor 4 antibodies), T2D: (type 2 diabetes)

## Abstract

The prevalence of obesity has risen in the last decades, and it has caused massive health burdens on people’s health, especially metabolic and cardiovascular issues. The risk of vitamin D insufficiency is increased by obesity, because adipose tissue alters both the requirements for and bioavailability of vitamin D. Exercise training is acknowledged as having a significant and long-term influence on body weight control; the favorable impact of exercise on obesity and obesity-related co-morbidities has been demonstrated via various mechanisms. The current work illustrated the effects of vitamin D supplementation and exercise on obesity induced by a high-fat diet (HFD) and hepatic steatosis in rats and explored how fatty acid transport protein-4 (FATP4) and Toll-like receptor-4 antibodies (TLR4) might be contributing factors to obesity and related hepatic steatosis. Thirty male albino rats were divided into five groups: group 1 was fed a normal-fat diet, group 2 was fed an HFD, group 3 was fed an HFD and given vitamin D supplementation, group 4 was fed an HFD and kept on exercise, and group 5 was fed an HFD, given vitamin D, and kept on exercise. The serum lipid profile adipokines, interleukin-6 (IL-6), and tumor necrosis factor-alpha (TNF-α) were analyzed, and the pathological changes in adipose and liver tissues were examined. In addition, the messenger–ribonucleic acid (mRNA) expression of FATP4 and immunohistochemical expression of TLR4 in adipose and liver tissues were evaluated. Vitamin D supplementation and exercise improved HFD-induced weight gain and attenuated hepatic steatosis, along with improving the serum lipid profile, degree of inflammation, and serum adipokine levels. The expression of FATP4 and TLR4 in both adipose tissue and the liver was downregulated; it was noteworthy that the group that received vitamin D and was kept on exercise showed also improvement in the histopathological picture of this group. According to the findings of this research, the protective effect of vitamin D and exercise against obesity and HFD-induced hepatic steatosis is associated with the downregulation of FATP4 and TLR4, as well as a reduction in inflammation.

## 1. Introduction

Obesity is a chronic condition characterized by excessive adipose tissue. It can be clinically controlled by lifestyle changes, pharmacological therapy, and surgery. An unhealthy body weight and abdominal adiposity, which reflects the amount of visceral fat mass, have been correlated with the development of chronic diseases, including type 2 diabetes mellitus (T2D), cardiovascular diseases, metabolic diseases, and cancers [1,2,3,4]. Investigations have demonstrated an increased incidence of cardiovascular illnesses in obese persons compared with lean persons with no metabolic risk factors [5].

The accumulation of adipose tissue, mainly white adipose tissue, which is regarded as the most essential fat depot, is linked to a metabolic state that is chronically pro-inflammatory at a low grade [6]. Adipocytes, macrophages, and T cells have been demonstrated to trigger inflammatory responses that disrupt the metabolic balance by secreting a variety of cytokines and adipokines in response to metabolic cues [7]. The size of adipocytes appears to be a significant influence on the formation of inflammatory cytokines such as interleukin-6 (IL-6), tumor necrosis factor alpha (TNF-α), and interleukin-1 beta (IL-1β) [8]. This low-grade inflammatory state is associated with insulin resistance, dyslipidemia, T2D, and cardiovascular disease [9].

Non-alcoholic fatty liver disease (NAFLD) is one of the obesity-related co-morbidities. NAFLD is often described as metabolic dysfunction-associated fatty liver disease (MAFLD) and is widely regarded as the most common form of liver disease in patients who abstain from drinking alcohol. The etiology of NAFLD is complex, with multiple variables implicated, including insulin resistance, obesity, and dyslipidemia [10,11,12]. Eating HFD over the long term is commonly associated with hepatic steatosis due to triglyceride (TG) accumulation and lipid dysregulation combined with increased oxidative stress and pro-inflammatory cytokines. In numerous rat models, HFD increased the body weight and adiposity because of its high-energy density [13]. 

Obesity, which raises the risk of vitamin D deficiency, might affect one’s vitamin D status. Adipose tissue is also a major factor in determining vitamin D needs and bioavailability. For many years, the association between obesity and the vitamin D status has been well known [14,15,16]. It has been observed in people of all races and sexes, as well as men and women of all ages [17]. Vitamin D deficiency may negatively impact glycemia, whereas vitamin D supplementation has been shown to restore normal glucose metabolism [18] and lower rates of metabolic syndrome [14]. A potentially non-pharmacological approach to avoiding obesity and weight gain is exercise training and improved physical activity. Exercise can reduce the deleterious effects of an HFD on adipose tissue, decrease inflammation in visceral white adipose tissue, and improve the lipid profile. Intermittent swimming exercise could decrease adiposity in HFD rats. Exercise could also decrease white adipose tissue inflammation in HFD model of obesity [19,20].

In the present study, we aimed to evaluate the effects of both vitamin D supplementation and swimming exercise on regulating obesity, adiposity, and hepatic steatosis. We also examined their effects on the serum lipid profile, degree of inflammation, adipokine serum levels, and expression of FATP4 and TLR4 in both adipose tissue and the liver.

## 2. Materials and Methods

### 2.1. Experimental Animals and Grouping

Eight-week-old male albino rats (n = 30) weighing 180–200 gm were utilized in the research. The rats were acquired from the rat’s animal breeding house of the Faculty of Medicine at Suez Canal University, Egypt. Rats were preconditioned and housed in five rat cages for 1 week at ambient temperature (22 ± 2 °C) with relative humidity (55 ± 10%) and a 12 h light/dark cycle. During this week, rats obtained water and a standard chow diet ad libitum.

### 2.2. Ethical Statement

The animal experiments were performed following the National Institutes of Health (NIH) ethical guidelines for the care and use of laboratory animals (NIH; Publication No. 85-23, revised 1985). This work was approved by the Ethical approval committee from the Suez Canal University (SCU) Animal Ethics Committee on 11 October 2020 (Protocol number: 4313). Before samples were collected, the animals were treated with ketamine/xylazine via an intraperitoneal injection, followed by cervical dislocation to minimize pain.

### 2.3. Diets

The Al-Gomhoureya Company provided the standard rat chow diet, which had 306.2 kcal per 100 grammes, 48.8 percent of its composition as carbohydrates, 21 percent of its composition as protein, and 3 percent of its composition as fat. The high-fat diet was modified in accordance with the description that was supplied by Levin and Dunn-Meynell (2002). The high-fat diet has a total caloric content of 414.0 kcal/100 g, of which 43.0 kcal come from carbohydrates, 17.0 kcal from protein, and 40.0 kcal come from fat. All the ingredients of the diet were mixed and prepared as pellets. The rat chow pellet represented 68% of the diet, while instant milk powder makes up 20%, maize oil makes up 6%, and ghee makes up 6% [21,22].

(Ghee is clarified butter; it’s more concentrated in fat than butter, as its water and milk solids have been removed.)

### 2.4. Experimental Design

#### 2.4.1. Groups

Thirty male albino rats were classified into the following groups:

CTL (normal control group) (n = 6): Control (normal diet): Rats were fed with a normal rat chow diet for 12 consecutive weeks.

HFD (high-fat diet group): (n = 6): Rats were supplied with a high-fat diet (HFD) for 12 weeks without treatment [23].

Vitamin D treated group: (n = 6) Rats were supplied with a HFD for 6 consecutive weeks, then received a concomitant therapeutic dose of vitamin D (500 IU/kg) by oral gavage for 6 weeks [24]. Vitamin D was purchased from Sigma Pharmaceutical Industries Agency, Nasr City, Egypt.

Swimming exercise treated group: (n = 6) Rats were supplied with a HFD for 6 consecutive weeks then underwent concomitant swimming exercise for 6 consecutive weeks (30 min/day) [25].

Combined (exercise+ vitamin D) treated group: (n = 6):

Rats were fed with HFD for 6 consecutive weeks, and then underwent concomitant daily swimming exercise (30 min/day) and vitamin D (500 IU/kg) by oral gavage for 6 consecutive weeks. 

#### 2.4.2. Swimming Exercise

The swimming training was accomplished in a glass water tank at 32 °C without a load, (dimensions: 100 cm × 40 cm × 60 cm). The water depth was adjusted to ensure the free swimming of rats. The period of the first swimming exercise was adjusted to 15 min, which was increased daily by 5 min to reach total time of 30 min. For 6 weeks, rats in the swimming exercise treated group and combined treated group swam for 30 min every day, five days a week [26].

### 2.5. Measurements of Obesity-Associated Parameters

#### Body Weight Gain 

An electric scale was used to determine the bodyweight of each rat in each group prior to the beginning of the investigation (Day 0), as well as after the conclusion of the treatment after 12 weeks. The body size was assessed at the end of the study by measuring the length from the nose to the anus. The following formula was utilized to estimate the Lee’s obesity index: Lee’s index = cube root of body weight (g)/body size (cm) [27].

### 2.6. Sample Collection

After a fasting period of 12 h, the rats were given an intraperitoneal injection of ketamine and xylazine to induce anesthesia at the conclusion of the 12th week of the program. Blood samples were taken from the heart. The serum was obtained by centrifuging the blood for 15 min at 800× *g* at 4 °C. After scarification, the liver was separated, weighed, and kept frozen at −80 °C.

### 2.7. Adiposity Index

Following the process of scarification, adipose tissue was removed from the epididymal, visceral, and retroperitoneal pad and then weighed. The adiposity index was calculated by taking the total fat weight of the epididymis, viscera, and retroperitoneum and dividing that number by the subject’s body weight multiplied by 100. The data were presented in the form of a percentage of total body fat [27]. 

### 2.8. Biochemical Analysis

#### 2.8.1. Lipid Profile 

The concentration of serum triglycerides (TGs) was assessed following the procedure of Fossati and Prencipe [28] using commercial kits(Cat. no. TR 20 30). The concentration of cholesterol in the serum was measured using a technique developed by Allian et al. [29] using commercial kits (Cat. no. CH 12 20)**.** The concentration of serum HDL-cholesterol was evaluated in accordance with the method of Burestein et al. [30] using commercial kits (Cat. no. CH 12 30). The concentration of serum LDL-cholesterol was measured following Wieland and Seidel [31] using commercial kits (Cat. no. CH 12 31). All kits were purchased from Biodiagnostic (El Omraniya, Egypt).

#### 2.8.2. Liver Enzymes

The serum levels of aspartate aminotransferase (AST), alanine aminotransferase (ALT), high-density lipoprotein cholesterol (HDL-C), and low-density lipoprotein cholesterol (LDL-C) were assessed utilizing an automatic analyzer (Olympus AU600, Tokyo, Japan) and proper commercial kits.

#### 2.8.3. Inflammatory Markers

Serum TNF-α and interleukin-6 levels were evaluated utilizing the rat TNF-α ELISA Kit (ab100784) and rat IL-6 ELISA Kit (ab100772), respectively, purchased from Abcam according to the manufacturer’s instructions.

#### 2.8.4. Hormones

##### Adipokines

Serum was collected in Eppendorf tubes and was used for measuring leptin, and ghrelin using a specific ELISA kit (Cat. No. EZRGRA and RAB0335, respectively, purchased from Sigma-Aldrich, St. Louis, MI, USA). The level of serum adiponectin was evaluated utilizing a rat Adiponectin ELISA (Cat. No. Ab239421, Abcam, Cambridge, UK), following the manufacturer’s instructions.

##### Insulin

Serum insulin concentration was determined utilizing insulin ELISA kit (Cat. No. ERINS, Invitrogen Bioservices India Pvt. Ltd., Bengaluru, India), according to the manufacturer’s instructions.

### 2.9. Quantitative Realtime PCR of FATP4 in Hepatic and Adipose Tissue

The RNeasy Mini Kit was used to extract RNA from hepatic and adipose tissue. (cat no 74104, Qiagen, Hilden, Germany). Reverse transcription followed by mRNA expression level by SYBR Green method (Cat. No. 208052, Qiagen, Germany). The sequence of the used primers is mentioned in (Table 1). The relative expression is being calculated according to Livak equation 2^−∆∆Ct^ [32].

### 2.10. Histological Analysis

The sections have been fixed with formalin (10%) and inserted into paraffin. From each block, 3 µm thickness sections were mounted to the slide of glass, discolored by hematoxylin and eosin (H&E) stain, and analyzed. Slides were subsequently scanned, and images were processed using the ImageJ scanner and viewer software (LOCI, University of Wisconsin, Madison, WI, USA).

#### 2.10.1. Histopathological Study of Liver Tissue Sections

Liver tissue sections were examined, and staging and grading of liver were graded according to the Supplementary Tables S1 and S2 [33].

#### 2.10.2. Histopathological Study of Adipose Tissue Sections

Adipose tissue sections were analyzed by determining the average number of adipocytes and each adipocyte area (µm^2^) in the field (40×). Adipocytes were evaluated in four different fields, and the average number and area were determined. Data were expressed as mean ± SD.

#### 2.10.3. Immunohistochemical Analysis

Selected paraffin blocks were cut into 4 µm thickness sections and were mounted to the glass slides. The sections were subsequently incubated with primary anti-Toll-Like receptor 4 (TLR4) antibodies (Anti-TLR4 antibody, ab22048; Abcam; Cambridge, UK), followed by the proper secondary antibody (Goat anti-Rabbit IgG (H + L) Secondary Antibody, HRP, Abcam; Cambridge, UK). All sections were counterstained for 30 s with hematoxylin before mounting and dehydration.

#### 2.10.4. Immunohistochemical Evaluation

The cells were considered positive if they exhibit a cytoplasmic reaction to TLR4 antibody. Modified Allred scoring system guidelines have been applied to semi-quantitatively assess the positive-stained sections [34]. The counts of the positive cells have been quantified in three different high-power fields (hpf). The scores of the positive cells’ percentage (0–5) and the cytoplasm staining intensity (0–3) were recorded to provide the final scores. The positive cells percentage was calibrated as follows: score 1-less than 10% positive cells; score 2- for 10–20% positive cells; score 3- for 20–50% positive cells; score 4- for 50–70% positive cells; and score 5-more than 70% positive cells. The SMA staining intensity was recorded as: score 1-weak; score 2-medium; and score 3-strong [34].

### 2.11. Statistical Analysis

The present work was a simple random allocation experimental study. Random allocation was done by using computer-generated random numbers.

The sample size was determined by using the equation of the difference between two means:

Sample size/group = 2 σ2 (Zα + Zβ)^2^/Δ^2^

where Zα: the value of standard normal distribution for type I error probability for two sided test (0.05/2) = 1.96. Zβ: The value of standard normal for the desired statistical power (90%) = 1.282. Δ: The lowest mean difference (u/mL) of HDL (as a one of lipid profile tests) between any two studying groups. σ: The within group standard deviation (u/ mL). 

Sample size/group = 2 σ 2 (Zα + Z β)^2^/∆^2^ = 2 × 0.05 × 2 (1.96 + 1.282)^2^/(0.85 − 0.26)^2^ = 1.296/0.14 = 6 rats/group [35] The calculated sample size was 6 rats/group. This sample size was included in the protocol of the study that was approved by the Ethical approval committee from the Suez Canal University (SCU) Animal Ethics Committee on 11 October 2020 (Protocol number: 4313).

The present work was a simple random allocation experimental study. Random allocation was done by using computer-generated random numbers. All data were statistically evaluated using a statistical package for the social sciences (SPSS) program (windows version number 22). All values were presented as means ± SD. The parametric data derived from three groups, or more were evaluated by one-way analysis of variance (ANOVA) followed by a post hoc Tukey’s test. The Non-parametric data, as well as discrete data from histologic scoring, were analyzed using the Kruskal–Wallis test followed by the Dunn test. Data were considered statistically significant with a *p*-value < 0.05.

## 3. Results

### 3.1. Measures of Adiposity

In this research, there was no discernible variation in the starting body weights of the animals across the various groups. When compared with the normal control group, the HFD group had a statistically significant rise in total body weight. The groups that were given vitamin D (D-group), kept on exercise (E-group), or given vitamin D and kept on exercise (combination group) demonstrated a significant decrease in the final body weight compared with the HFD group. As indicated in Table 1, the combination group had a significant reduction in final body weight compared with the normal control group, and the reduction was non-significant compared with the D-group and E-group.

The HFD group had a considerably larger body size compared with the normal control group. Conversely, the E-group had a significantly smaller body size than the HFD group. The difference in body size among the D-group, combination group, and HFD group was not statistically significant. The E-group showed a significant reduction in body size compared with the control group (see Table 2).

The HFD group demonstrated a statistically significant increase in fat weight compared with the normal group. When compared with the HFD group, the D-group, E-group, and combination group all had a substantial reduction in fat weight. The fat weight of the E-group demonstrated a non-significant difference compared with the D-group and the combination group (see Table 2).

The adiposity index for the HFD group was significantly greater than for the regular control group. The D-group, E-group, and combination group all had a statistically significant decrease in the adiposity index compared with the HFD group. The E-group exhibited the greatest decrease in the adiposity index; however, this decrease was not statistically significant in comparison with the D-group and combination group (*p* = 0.81 and 0.89, respectively). When comparing the various groups, there were no discernible variations in the Lee’s obesity index of any of the animals (see Table 2).

### 3.2. Lipid Profile

The HFD group had a significant increase in low-density lipoprotein (LDL), very low density lipoprotein (VLDL), serum total cholesterol, and TG levels compared with the control group. The D-group, E-group, and combination group had a significant decrease in LDL, VLDL, serum total cholesterol, and TG levels in comparison with the HFD group. The combination group had a non-significant decrease in the serum total cholesterol level and VLDL level in comparison with the difference normal control group. The combination group had a significant reduction in the serum total cholesterol level in comparison with the D-group and E-group. Further, the combination group demonstrated a significant decrease in VLDL levels compared with the D-group and a non-significant reduction in comparison with the E-group. Regarding LDL levels, the combination group showed a significant reduction when compared with the control group. The reduction was significant in comparison with the D-group and E-group. Regarding TG levels, the combination group exhibited a significant decrease compared with the control group, D-group, and E-group (Table 3).

In comparison with the control group, the HFD group demonstrated a statistically significant decrease in the blood high-density lipoprotein (HDL) level. The D-group, E-group, and combination group demonstrated a substantial upregulation in the serum HDL level. In comparison with the HFD group, the D-group and E-group exhibited a substantial increase in blood HDL levels. Nevertheless, the combination group had a non-significant upregulation in HDL levels. At the same time, the difference was significant compared with the control group (see Table 3).

### 3.3. Liver Enzymes Levels

The serum aspartate transferase (AST) and alanine transaminase (ALT) concentrations were measured to see if vitamin D and exercise might prevent HFD-induced liver damage. 

In the HFD group, both the ALT and AST levels showed a significant elevation in comparison with the control group. Regarding the ALT, the D-group exhibited a non-significant decrease in comparison with the HFD group. In contrast, the E-group and combination group showed a significant reduction in comparison with the HFD group. The combination group demonstrated a significant decrease in comparison with the D-group and a non-significant reduction in comparison with the control group and E-group (Table 4).

Regarding the AST, the D-group revealed a significant decrease in comparison with the HFD group. The E-group and combination group showed significant reductions in comparison with the HFD group. In comparison with the D-group and combination group, the reduction was statistically significant. On the other hand, the reduction was not statistically significant compared with the control group, E-group, and combination group (see Table 4).

### 3.4. Inflammatory Markers Levels

**Figure 1:**
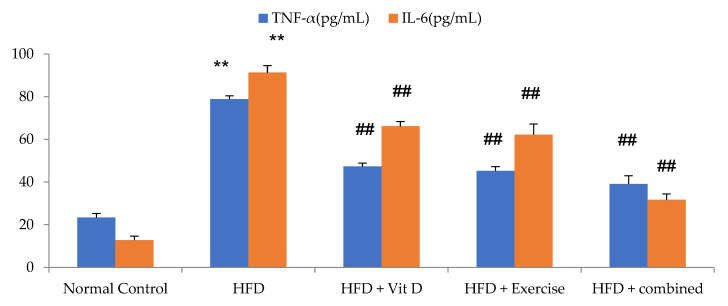
Inflammatory markers of the studied groups, including TNF-α and IL-6. Values are expressed as mean ± SD, statistically significant difference, ** *p*, ## *p* ˂ 0.01, in comparison to the control group, (#) in comparison to HFD.

In our investigations, the impact of an HFD on levels of inflammatory markers was assessed. The HFD group exhibited upregulation in levels of the inflammatory markers TNF-α and IL-6 compared with the control group. In comparison with the HFD group, serum levels of TNF-α and IL-6 were significantly lower in the D-group, E-group, and combination group. When compared with each other, individual modality treatment was associated with a non-significant difference regarding serum IL-6 and TNF-α. However, the combination group had a substantial reduction in serum TNF-α and IL-6 compared with the HFD group and E-group (Figure 1).

### 3.5. Adipokines Levels 

The circulating levels of adipokines in the HFD group were measured; there was a significant reduction in levels of ghrelin and serum adiponectin but a significant increase in levels of serum leptin in comparison with the control group. The D-group, E-group, and combination group showed a significant rise in serum adiponectin and ghrelin levels and a decrease in serum leptin levels compared with the HFD group (*p* < 0.01). The difference in adiponectin, ghrelin, and leptin levels between individual treatment modalities was non-significant (*p* = 0.23, 0.69, 0.42, respectively). When compared with the D-group and E-group, the combination group exhibited a statistically significant elevation in serum adiponectin levels. 

**Figure 2:**
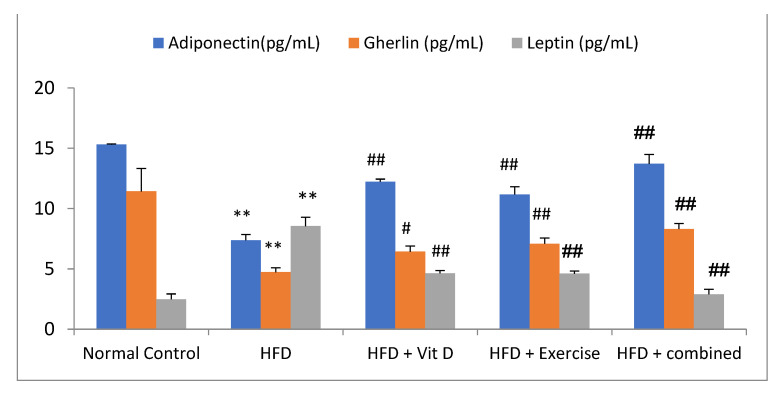
Adipokines levels of the studied groups include adiponectin, ghrelin, and leptin. Data expressed as mean ± SD, statistically significant difference, ** *p*, ## *p* ˂ 0.01, and # *p* ˂ 0.05 (*) in comparison to the control group, (#) in comparison to HFD.

Regarding ghrelin, the elevation of it in the combination group was significant compared with the D-group, while it was non-significant compared with the E-group (*p* = 0.13). The reduction in leptin levels in the combination group was significant compared with the D-group and E-group (Figure 2).

### 3.6. Insulin Level

**Figure 3:**
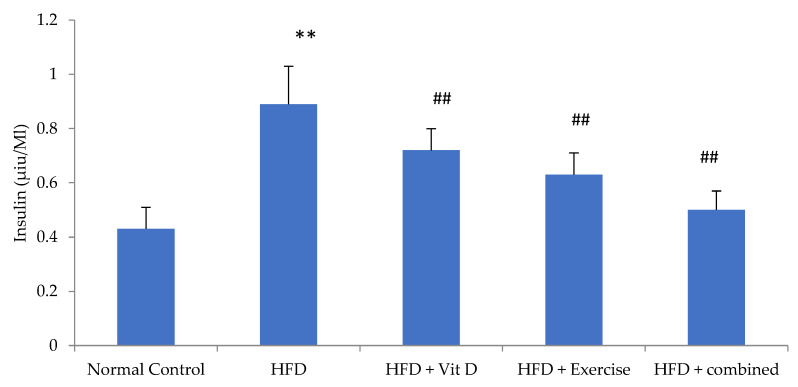
Insulin levels of the studied groups. Data expressed as mean ± SD, statistically significant difference, ** *p*, ## *p* ˂ 0.01 in comparison to the control group, (#) in comparison to HFD.

Obesity has been correlated with insulin resistance and elevations in insulin levels. In the present investigation, those who followed the HFD diet had a statistically significant higher level of blood insulin in comparison with those who followed the usual control diet. In comparison with the HFD group, the levels of blood insulin in the D-group, E-group, and combination group were substantially decreased. The combination group showed an insignificant decrease in the serum insulin level in comparison with the normal control group and E-group. In addition, the decrease was considerable in comparison with the D-group (Figure 3). 

### 3.7. Expression of FATP4 in Tissues

**Figure 4:**
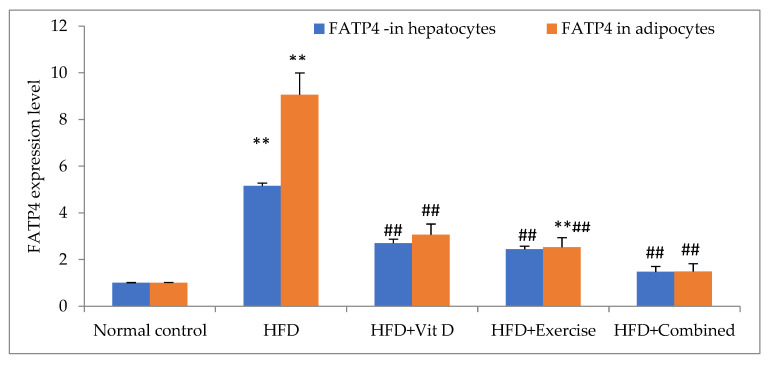
FATP4 expression level in hepatocytes and adipocytes in the studied groups. Data expressed as mean ± SD, statistically significant difference, ** *p*, ## *p* ˂ 0.01, in comparison to the control group, (#) in comparison to HFD.

The HFD group showed a significant increase in FATP4 mRNA expression levels in liver and adipose tissues as compared with the control group. The D-group, E-group, and combination group exhibited a significant decrease in FATP4 mRNA expression levels in liver and adipose tissues as compared with the HFD group. The combination group demonstrated a significant downregulation in liver tissue levels of FATP4 mRNA expression in comparison with the control group, D-group, and E-group. In adipose tissue, a non-significant downregulation in FATP4 expression was seen in the combination group in comparison with the control group and a significant downregulation was seen in comparison with the D-group and E-group (Figure 4).

### 3.8. Histopathological Evaluation of Adipose Tissue and Liver Tissues Sections

**Figure 5:**
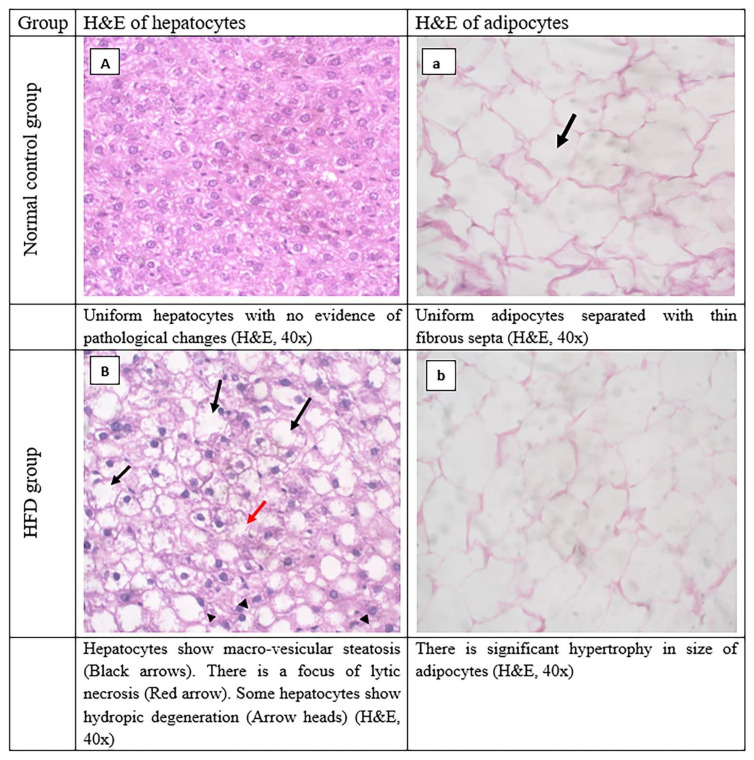

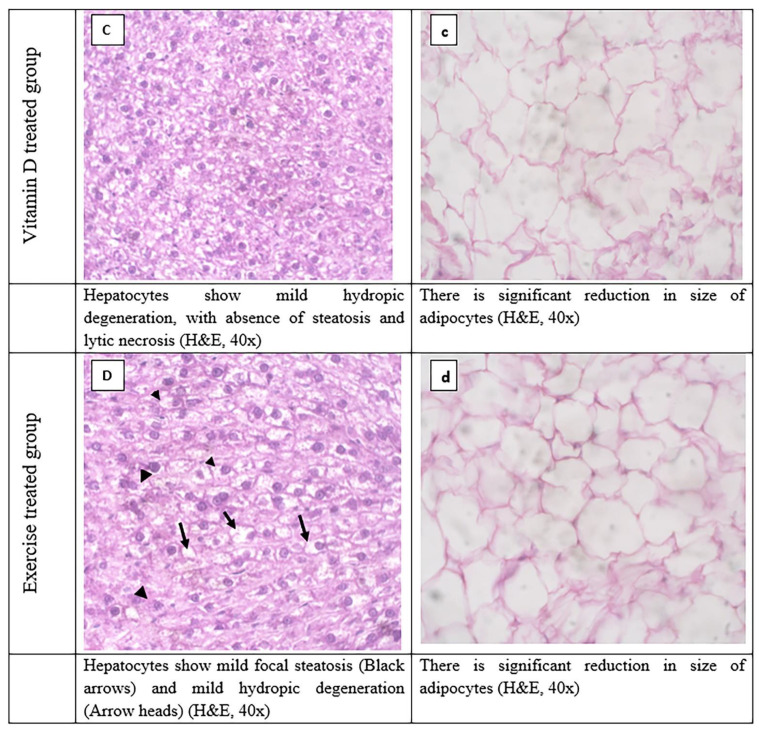

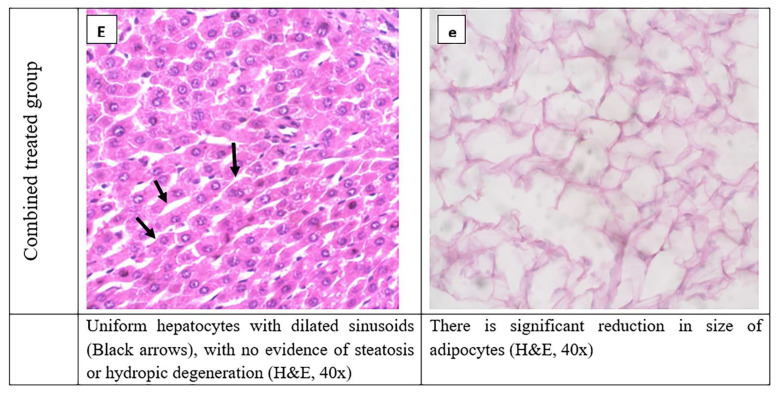
Histopathological evaluation of liver and adipose tissue sections.

The adipose tissue, in the control group showed uniform adipocytes separated with thin fibrous septa. The HFD group exhibited a significant elevation in the size of adipocytes as compared with the normal group. The D-group and E-group showed a significant reduction in the size and number of their adipocytes compared with the HFD group. The combination group had a significant reduction in the size of its adipocytes compared with the HFD group and control group, as shown in Figure 5A–E and Table 5.

Regarding liver tissue, the control group showed normal liver tissue and uniform hepatocytes with no evidence of pathological changes. There were no fatty changes, no lytic necrosis, and no hydropic degeneration (Figure 5A, Table 6). In the HFD group, there was evidence of pathological changes in the liver tissue, such as multiple foci of lytic necrosis, marked fatty changes, and marked hydropic degeneration (see Figure 5B, Table 6). The D-group had uniform hepatocytes with mild fatty changes, mild lytic necrosis, and hydropic degeneration (see Figure 5C, Table 6). The E-group showed uniform hepatocytes with mild fatty changes, mild lytic necrosis, and hydropic degeneration (see Figure 5D, Table 6). The combination group had normal liver tissue and uniform hepatocytes with no evidence of pathological changes. There were no fatty changes, no lytic necrosis, and no hydropic degeneration (see Figure 5E, Table 6).

### 3.9. Immunohistochemical Evaluation of Adipose Tissue and Liver Tissues Sections

**Figure 6:**
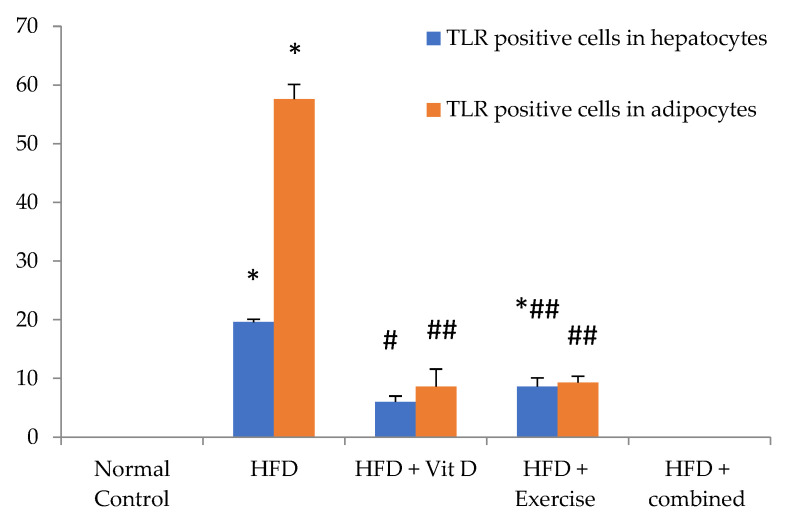
Level of TLR expression by immunohistochemistry in the studied groups in hepatocytes and adipocytes. Values are expressed as mean ± SD, statistically significant difference, ## *p* ˂ 0.01, and * *p*, # *p* ˂ 0.05 (*) compared to normal control group, (#) compared to HFD. HDF = high fat diet.

**Figure 7:**
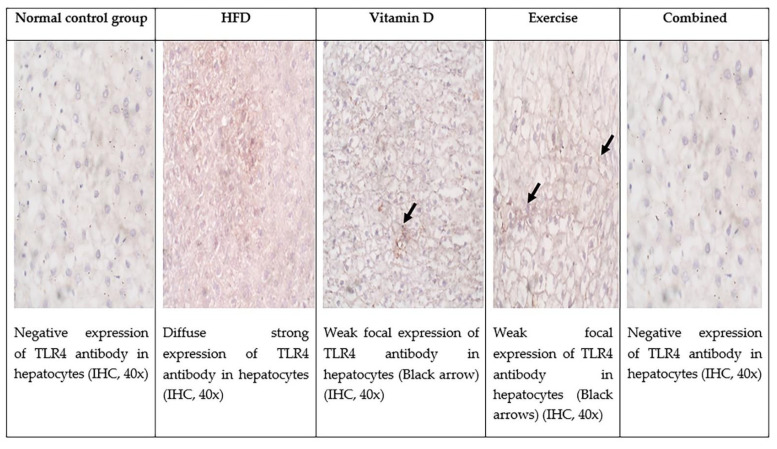
TLR expression by immunohistochemistry in the five studied groups in hepatocytes.

**Figure 8:**
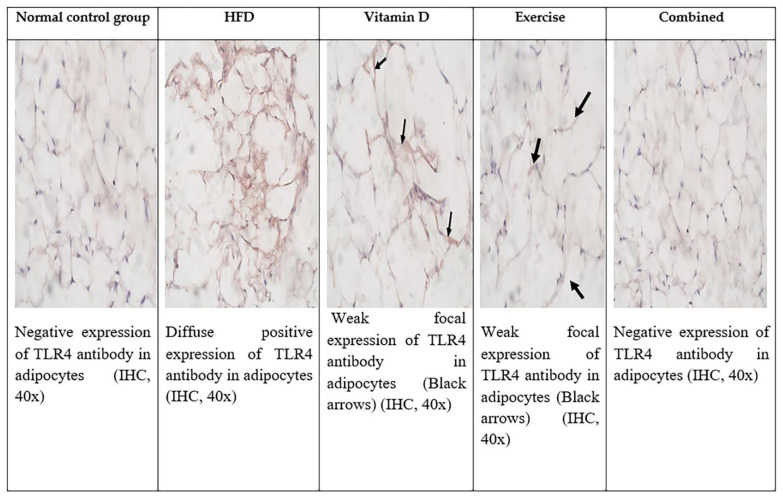
TLR expression by immunohistochemistry in the five studied groups in adipocytes.

Immunohistochemical staining of TLR4 in adipose tissue and liver revealed that there was negative expression of TLR4 in adipose tissue and liver of control group while in HFD group there was significant diffuse positive expression. The D-group and E-group had a significant decrease in the expression of TLR4 in both liver and adipose tissue. The combined treatment group showed negative expression of TLR4 in both liver and adipose tissue as shown in Figure 6, Figure 7 and Figure 8 and Table 7.

## 4. Discussion

Obesity is associated with several life-threatening conditions and is linked to increased mortality and morbidity rates. An HFD, in general, promotes obesity-related co-morbidities such as metabolic syndrome, which encompasses a pro-inflammatory and pro-thrombotic state, atherogenic dyslipidemia, cardiovascular disease, high blood pressure, oxidative stress, and central obesity [36,37,38]. NAFLD is one of the obesity-related co-morbidities. It is associated with many liver disorders, including cirrhosis, severe fibrosis, nonalcoholic steatohepatitis (NASH), and simple steatosis [10,11,39].

Vitamin D and exercise are beneficial in obesity management, but the potential mechanistic relevance of exercise and supplementation of vitamin D in obesity and obesity-related co-morbidities is now being contested. As a result, this research was performed to explore their effect on FATP4 and TLR4 in adipose tissue and liver tissue in a rat model of HFD-induced obesity.

The major findings of the present study are summarized in the subsequent points. (1) HFD was associated with increased body weight and significant elevation in the profiles of lipids, including TG, total cholesterol, LDL, VLDL cholesterol, and HDL cholesterol, along with the elevation of inflammatory markers. Furthermore, there was increased expression of FATP4 and TLR4 in adipose tissue and liver tissue in HFD-fed rats, which was associated with significant hypertrophy in the size of adipocytes and fatty degeneration of the liver hepatocytes, demonstrating steatosis. (2) The D-group, E-group, and combination group showed improvement in the findings as mentioned earlier compared with the HFD group with decreased FATP4 and TLR4 expression. (3) The combination group outperformed the individual modalities, with a more pronounced decrease in FATP4 and TLR4 expression associated with improvement in fatty liver degeneration and adipocyte mass. 

Dysregulation of adipose fatty acid metabolism is a substantial factor in developing obesity and related metabolic diseases. This imbalance is primarily caused by differences in the rates of long-chain fatty acid (LCFA) influx, export, and metabolism by adipose tissue [40,41,42]. The FATP family is made up of six members (FATP1 to 6), with FATP4 displaying tissue-specific expression in the liver, skin, brain, adipose tissue, heart, and skeletal muscle [43]. It is the only FATP located in the small intestine [44,45]. The physiological role of FATP4 in most of these tissues is unknown. Multiple investigations found an enhanced cellular inflow of fatty acids to result from overexpression of FATP4 in several cultured cell lines [46,47,48]. FATP4 was first thought to be a transmembrane transport protein [49].

Evidence suggests that FATP4 is confined to the endoplasmic reticulum and other intracellular compartments, implying that FATP4 may play an essential role in intracellular LCFA processing [48,50,51]. FATP4 has acyl-CoA synthetase activity, which allows it to catalyze and activate the fatty acid metabolism (most likely the synthesis of phospholipids and TGs) [44]. The enzymatic activity of FATP4 has been correlated with vectorial acylation (i.e., the triggering of lipid uptake [50]), which indirectly increases LCFA uptake. These LCFAs are subsequently utilized for energy production and cellular respiration. As the quantity of intracellular free LCFAs decreases, the cell takes up more LCFAs [52,53]. 

Adipocytes with FATP4-knockdown revealed increased basal lipolysis, implying a function in the esterification of fatty acid in 3T3-L1 adipocytes. Because of their different cellular locations and activities, FATP4 and FATP1 influence the size of TG lipid droplets and other complex lipid pools. These activities have been linked to insulin resistance and obesity development, indicating that FATP4 may act in adipocytes through LCFA re-esterification following lipolysis [40]. FATP4 expression was upregulated in acquired obesity, regardless of genetic variables. Furthermore, expression levels of CD36 and FATP4 were linked to obesity and insulin resistance. This was compatible with our finding regarding FATP4 increased expression and elevated lipid profile indices in the current study [54].

FATP4 has been linked to the development of NAFLD by findings showing that increased FATP4 leads to the dysregulation of fatty acids [55]. Previous work regarding the contribution of CD36 and FATP4 to hepatic LCFA uptake as a possible player in NAFLD or NASH found that overexpression of FATP2 and FATP4 in cells was associated with elevated oleate uptake in comparison with control cells. Free LCFA concentrations in the intracellular and extracellular domains could be created as a result of the proposed enhanced enzyme activity; this reduced the intracellular pool of free LCFAs and affirmed that at least FATP2 and FATP4 had a significant impact on the uptake of LCFAs [56]. FATP4 expression was elevated in the adipose tissue and liver tissue of the HFD group, as shown in the current study.

TLR4 represents the link between fatty acids, immunity, and inflammation and has been linked to the pathogenesis of obesity, insulin resistance, and NAFLD. Obesity-induced inflammation is mostly triggered by the TLR4 signaling pathways, and it leads to the production of TNF-α pro-inflammatory cytokines. Activation of the TLR4 signaling pathway has been implicated in alcoholic and non-alcoholic liver disease and is regarded as the major pathway for the development of NAFLD and NASH [57,58,59,60,61].

In the current study, the applied model provided the typical features of NASH in humans. In this regard, it was shown that there was a substantial increase in dyslipidemia and body weight and amplified liver function, together with the dysfunction of adipocytes and the increase of serum TNF-α and leptin and a noticeable decrease in adiponectin levels and elevated expression of TLR4 in the adipose tissue and liver. FATP4 and TLR4 might be employed as possible medications for obesity and other obesity-related conditions, such as steatosis of the liver. To the best of our knowledge, this is the first study to show the effect of vitamin D and exercise on obesity and one of the obesity-related liver diseases in terms of the influence of FATP4.

Vitamin D impacts several organic processes, including adipose tissue morphological function and lipid metabolism. Obesity reduces vitamin D bioavailability. Previous research on the causal relationship between obesity and vitamin D deficiency (VDD) discovered that VDD causes obesity and has been connected to hyperglycemia, hyperinsulinemia, insulin resistance, impaired β-cell function, and elevated homeostatic model assessment for insulin resistance (HOMA-IR) and intrahepatic lipid concentrations. Vitamin D has a beneficial effect on insulin production, insulin receptor expression, and insulin sensitivity. It enhances signal transduction by modulating extracellular calcium, boosting insulin-dependent glucose oxidation, and decreasing systemic inflammation. In obese people and animals, vitamin D improved HDL cholesterol levels while lowering hypertriacylglycerolemia levels in adipose tissue [62,63,64,65,66,67]. 

Vitamin D therapy reduced body weight gain and abdominal fat deposition, while it enhanced the plasma lipid profile and insulin sensitivity in obese rats that had been fed a Western diet. As demonstrated in our work, these effects were followed by decreased leptin and circulating TNF-α levels [48]. Pro-inflammatory adipokines IL-6 and TNF-α are produced and released when vitamin D deficiency occurs, resulting in an increase in the amount of fat in the body. Vitamin D deprivation enhances the expression of adipogenic genes and reduces the extent of mRNA for some genes involved in the metabolism of fatty acids including carnitine palmitoyl transferase1 (CPT1), peroxisome proliferator-activated receptor-gamma coactivator-1alpha (PGC1), very long chain acyl-CoA dehydrogenase (VLCAD), peroxisome proliferator-activated receptor (PPAR), and long-chain acyl-CoA dehydrogenase, in addition to reducing AMPK/SIRT1 activity [54]. 

Previous studies suggested that vitamin D reduces TG levels and lipid droplet accumulation in NAFLD produced by an HFD. Additionally, it lowers high levels of liver enzymes in a mechanistic manner by reducing lipogenesis, increasing lipid oxidation, mitigating the effects of oxidative stress, and lowering pro-inflammatory marker levels. There was a reduction in the expression of SREBP-1c and its target genes, fatty acid synthase and acetyl CoA carboxylase, which demonstrated that vitamin D increased the production of antioxidant and anti-inflammatory chemicals. Simultaneously, it triggered the upregulation of hepatic mRNA expression of PPARα and CPT-1 and reduced leptin, induced adiponectin/AdipoR1 with suppression of the p53–p21 pathway of caspase-3/apoptosis, and increased SMP-30 levels [68,69,70,71,72].

Regarding the effect of exercise on obesity and obesity-related co-morbidities, it is well documented that the mechanism underlying this beneficial effect is still under debate. Exercise improves lipid profiles and enhances insulin sensitivity. In a trial to uncover the mechanism behind the beneficial effect of exercise, Jordy and colleagues performed lipidomic profiling in the skeletal muscle and liver of exercise-trained mice after the induction of insulin resistance and obesity by high-fat feeding for 4 weeks. They found that the exercise decreased diacylglycerol and cholesterol esters in the livers of trained mice. Further, an increased ratio of phosphatidylcholine (PC) to phosphatidylethanolamine (PE) was correlated with membrane integrity and associated with hepatic disease progression. A concomitant decrease was noted for the liver’s fatty acid transporters FATP4 and CD36 [73]. This was coordinated with our finding regarding FATP4 expression in the liver. To the best of our knowledge, there are no available data on the effect of exercise on the expression of FATP4 in adipose tissue. However, exercise may have its beneficial effect by reducing such expression, as reported in the current study. Our results suggested that exercise promotes a coordinated decrease in the entry of fatty acid into hepatocytes and adipose tissue. In lipid accumulation at ectopic sites, involving the liver and skeletal muscle, adipose tissue is a common result of obesity.

One interesting study regarding the effect of exercise on the expression of FATP4 in skeletal muscles revealed that training increased the protein content of FATP4 by 33%, associated with enhanced lipid oxidation during prolonged submaximal exercise [74]. Thus, the increased fuel demand induced by exercise training in skeletal muscles could be another benefit of exercise, through reducing the uptake of LCFA by the adipose tissue and liver to decrease TG formation and lipid accumulation while increasing expression in muscle to increase uptake and oxidation. 

In agreement with our findings, several investigations found that inhibiting the activity of the TLR4/NF-kB signaling pathway and gene expression led to a reduction in the activity of TNF-α, IL-6, and other inflammatory mediators [75,76]. In obese rats, vitamin D deprivation has been demonstrated to increase the expression levels of TLR2, TLR4, and TLR9 hepatic mRNA [77]. Supplementation of vitamin D has been indicated to reduce TLR4 expression in the ileum of an obese mouse model of NASH [78,79]. Exercise has been shown to reduce TLR4 expression in rats with diet-induced obesity, with both acute and chronic exercise causing a significant suppression in the TLR4 signaling pathway in the muscle, liver, and adipose tissue and improvement of insulin signaling and sensitivity [80,81]. 

## 5. Conclusions

In conclusion, we have presented evidence that expression levels of FTAP4 in liver and visceral adipose tissue are associated with acquired obesity, obesity-associated liver disease, and insulin resistance in rat. Obesity increased FATP4 expression. Following the induction of obesity, vitamin D supplementation and exercise attenuated body weight gain and abdominal adiposity, and NAFLD. Further, it ameliorated the plasma lipid profile in obese rats. These effects may be mediated, at least in part, by decrease of FTAP4, TLR4 expression with a reduction of circulating levels of leptin and TNF-α. When considered as a whole, the findings of this study lend credence to the hypothesis that a combination of vitamin D and physical activity could be an effective therapy of obesity and the comorbidities that are associated with it.

## Figures and Tables

**Table 1:** The primer sequences of RT-PCR of hepatic and adipose tissue FATP4.

Primer	Sequence
FATP4	Forward: 5′GCACTCTCACTTCCTCTTGC3′ Reverse: 5′GCAGGAAAGGAACAATGCCA3′
GAPDH	Forward: 5′CCCCATTTGATGTTAGCGGG3′ Reverse: 5′AACGACCCCTTCATTGACCT3′

The primer was designed using primer3web version 4.1.0 & NCBI and tested in silico using Sequence manipulation suite (SMS) version 2, PCR Primer Stat tool & UCSC In silico PCR and in silico PCR amplification tool.

**Table 2:** Effects of vitamin D, swimming exercise, combined (vitamin D + exercise), and HFD on body weight, body size, fat weight, adiposity index and Lee’s index.

Group Variable (Mean ± SD)	Normal Control	HFD	Vitamin D	Exercise Treated	Combined
Initial weight (gm)	232.5 ± 19.48	210 ± 22.36	219.17 ± 23.54	224.17 ± 18	219 ± 22.74
Final weight (gm)	249.16 ± 18.28	418.57 ± 31.72 **	363.33 ± 13.29 #	339 ± 30 ##	312 ± 13.4 *##
Body size (cm)	22.83 ± 1.72	27 ± 1.15 **	26.16 ± 1.33	24.33 ± 1.03 #	25.8 ± 0.84 **
Fat weight(gm)	0.5 ± 0.5	20.57 ± 5.44 **	11.42 ± 2.53 ##	9.08 ± 1.02 ##	9.70 ± 0.84 ##
Adiposity Index	0.21 ± 0.22	4.9 ± 1.27 **	3.16 ± 0.78 ##	2.70 ± 0.30 ##	3.11 ± 0.24 ##
Lee’s obesity index	0.27 ± 0.02	0.28 ± 0.02	0.27 ± 0.01	0.29 ± 0.01	0.26 ± 0.02

Data presented as mean ± SD, statistically significant difference, ** *p*, ## *p* ˂ 0.01 & * *p*, # *p* ˂ 0.05 (*) compared to the normal control group, (#) compared to HFD. HFD = high fat diet.

**Table 3:** Illustrates the lipid profile results of the different groups, including total cholesterol, HDL, LDL, VLDL, and triglycerides.

Group Variable (Mean ± SD)	Normal Control	HFD	Vitamin D	Exercise	Combined
Total cholesterol (mg/dL)	56.70 ± 6.01	231.40 ± 33.28 **	115.83 ± 21.54 ##	97.50 ± 15.41 ##	64.29 ± 10.18 ##
HDL (mg/dL)	52.50 ± 9.35	17.14 ± 3.93 **	29.16 ± 3.76 #	32 ± 3.76 #	40 ± 10.81 *##
LDL (mg/dL)	59.90 ± 12.50	149.14 ± 8.89 **	122.83 ± 3.59 ##	112.97 ± 4.95 ##	93.71 ± 5.6 **##
VLDL (mg/dL)	29.50 ± 3.72	47.43 ± 3.10 **	38 ± 1.87 ##	35.17 ± 1.10 ##	31.50 ± 3.35 ##
TG (mg/dL)	140.50 ± 5.71	207.85 ± 9.11 **	190 ± 2.82 ##	177.67 ± 2.80 ##	167.14 ± 4.80 ##**

Data expressed as mean ± SD, statistically significant difference, ** *p*, ## *p* ˂ 0.01 & * *p*, # *p* ˂ 0.05 (*) compared to the normal control group, (#) compared to HFD. HFD = high fat diet.

**Table 4:** Illustrates the liver enzymes, ALT, and AST of the studied groups.

Group Variable (Mean ± SD)	Normal Control	HFD	Vitamin D	Exercise	Combined
ALT (mg/dL)	19.8 ± 5.80	31.4 ± 5.20 **	26.80 ± 2.20	21.20 ± 1.92 ##	18 ± 3.80 ##
AST (mg/dL)	25.60 ± 7.20	39.80 ± 1.50 **	31.40 ± 3.00 #	27.6 ± 2.40 ##	20.8 ± 2.77 ##

Data expressed as mean ± SD, statistically significant difference, ** *p*, ## *p* ˂ 0.01 & # *p* ˂ 0.05 (*) compared to the normal control group, (#) compared to HFD. HFD = high fat diet.

**Table 5:** Histopathological evaluation of adipose tissue sections.

Group Variable (Mean ± SD)	Normal Control	HFD	Vitamin D	Exercise	Combined
Size of adipocytes (mm^2^)	647.75 ± 70.9	950.7 ± 154.6 *	634 ± 22.6 ##	465.7 ± 186.8 ##	436.2 ± 91.8 *##
Number of adipocytes	57.2 ± 6	62.2 ± 6.8	51.5 ± 1.2 ##	53 ± 2.4 ##	47.5 ± 2 **##

Data expressed as mean ± SD, statistically significant difference, ** *p*, ## *p* ˂ 0.01, and * *p* ˂ 0.05 (*) in comparison normal control group, (#) compared to HFD.

**Table 6:** Histopathological evaluation of liver tissue sections.

Group Variable (Mean ± SD)	Normal Control	HFD	Vitamin D	Exercise	Combined
Liver tissue Grading	0	1 (One focus of lytic necrosis) Marked fatty change, Marked hydropic degeneration	0 No fatty changesMild hydropic degeneration	0 Mild fatty change, Mild hydropic degeneration	0

**Table 7:** Immunohistochemical scoring for TLR4 expression in adipocytes and hepatocytes.

Group Variable (Mean ± SD)	Normal Control	HFD	Vitamin D	Exercise	Combined
TLR positive cells in hepatocytes	0	19.6 ± 0.5 **	6 ± 1 ##	8.6 ± 1.5 *##	0 ##
Staining intensity	0	Moderate Score 2	Weak Score 1	Weak Score 1	0
Total score	0	4	2	2	0
Group variable (mean ± SD)	Normal group	HFD group	Vitamin D treated group	Exercise treated group	Combined treated group
TLR positive cells in adipocytes	0	57.6 ± 2.5 **	8.6 ± 3 ##	9.3 ± 1.1 ##	0 ##
Staining intensity	0	Strong Score 3	Weak Score 1	Weak Score 1	0
Total score	0	7	2	2	0

Data expressed as mean ± SD, statistically significant difference, ** *p*, ## *p* ˂ 0.01, and * *p* ˂ 0.05 (*) in comparison to the control group, (#) in comparison to HFD.

## Data Availability

Data is contained within the article.

## Supplementary Materials

The following supporting information can be downloaded at: https://www.mdpi.com/article/10.3390/ijerph192113740/s1. Table S1. Modified liver staging: architectural changes, fibrosis, and cirrhosis. Table S2. Modified liver histological activity index (HAI) grading: necro-inflammatory scores.
