# Supplementary material for: Vitamin D and Swimming Exercise Prevent Obesity in Rats under a High-Fat Diet via Targeting FATP4 and TLR4 in the Liver and Adipose Tissue
Source: Int J Environ Res Public Health. 2022 Oct 22;19(21):13740. doi: 10.3390/ijerph192113740 (PMC9656563; doi:10.3390/ijerph192113740)
Supplement: Supplementary file 1 [file ijerph-19-13740-s001.zip › ijerph-1834233-supplementary.pdf]

Table S1. Modified liver staging: architectural changes, fibrosis, and cirrhosis.

Score	Lobular architecture
0	Normal (absence of fibrosis)
1	Fibrous expansion of some portal areas
2	Fibrous expansion of most portal areas, with portal-portal septa
3	Fibrous extension of portal spaces with portal-portal and portal-central septa, with possible nodule formation
4	Cirrhosis with predominant nodular areas in relation to the remaining lobules

Table S2. Modified liver histological activity index (HAI) grading: necro-inflammatory scores.

A. Periportal or periseptal interface hepatitis (piecemeal necrosis)	Degree
Absent	0
Mild (focal, few portal areas)	1
Mild/moderate (focal, most portal areas)	2
Moderate (continuous around 60% of tracts or septa)	3
Severe (continuous around >50% of tracts or septa)	4
**B. Confluent necrosis**	
Absent	0
Focal confluent necrosis	1
Zone 3 necrosis in some areas	2
Zone 3 necrosis in most areas	3
Zone 3 necrosis+ occasional portal-central (P-C) bridging	4
Zone 3 necrosis+ multiple P-C bridging	5
Panacinar or multiacinar necrosis	6
**C. Focal (spotty) lytic necrosis, apoptosis, and focal inflammation**	
Absent	0
One focus or less per 10X objective	1
Two to four foci per 10X objective	2
Five to ten foci per 10X objective	3
More than ten foci per 10X objective	4
**D. Portal inflammation**	
Absent of portal lymphocytes	0
Mild number of portal lymphocytes	1
Moderate number of portal lymphocytes	2
Marked number of portal lymphocytes	3
Strongly marked number of portal lymphocytes	4
